# The Contrasting Role of p16^Ink4A^ Patterns of Expression in Neuroendocrine and Non-Neuroendocrine Lung Tumors: A Comprehensive Analysis with Clinicopathologic and Molecular Correlations

**DOI:** 10.1371/journal.pone.0144923

**Published:** 2015-12-16

**Authors:** Nicola Fusco, Elena Guerini-Rocco, Alessandro Del Gobbo, Renato Franco, Federica Zito-Marino, Valentina Vaira, Gaetano Bulfamante, Giulia Ercoli, Mario Nosotti, Alessandro Palleschi, Silvano Bosari, Stefano Ferrero

**Affiliations:** 1 Division of Pathology, Fondazione IRCCS Ca’ Granda—Ospedale Maggiore Policlinico, Milan, Italy; 2 Department of Pathology, Istituto Nazionale Tumori—IRCCS Fondazione Pascale, Naples, Italy; 3 Istituto Nazionale Genetica Molecolare “Romeo ed Enrica Invernizzi”, Milan, Italy; 4 Department of Pathophysiology and Organ Transplantation, University of Milan, Milan, Italy; 5 Division of Pathology, San Paolo Hospital; Department of Health Sciences, University of Milan, Milan, Italy; 6 Division of Thoracic Surgery, Fondazione IRCCS Ca’ Granda, Ospedale Maggiore Policlinico, Milan, Italy; 7 Department of Biomedical, Surgical and Dental Sciences, University of Milan, Milan, Italy; University of Naples Federico II, Naples, ITALY

## Abstract

Lung cancer encompasses a constellation of malignancies with no validated prognostic markers. p16^Ink4A^ expression has been reported in different subtypes of lung cancers; however, its prognostic value is controversial. Here, we sought to investigate the clinical significance of p16^Ink4A^ immunoexpression according to specific staining patterns and its operational implications. A total of 502 tumors, including 277 adenocarcinomas, 84 squamous cell carcinomas, 22 large cell carcinomas, 47 typical carcinoids, 12 atypical carcinoids, 28 large cell neuroendocrine carcinomas, and 32 small cell carcinomas were reviewed and subjected to immunohistochemical analysis for p16^Ink4A^ and Ki67. The spectrum of p16^Ink4A^ expression was annotated for each case as negative, sporadic, focal, or diffuse. Expression at immunohistochemical level showed intra-tumor homogeneity, regardless tumor histotype. Enrichments in cells expressing p16^Ink4A^ were observed from lower- to higher-grade neuroendocrine malignancies, whereas a decrease was seen in poorly and undifferentiated non-neuroendocrine carcinomas. Tumor proliferation indices were higher in neuroendocrine tumors expressing p16^Ink4A^ while non-neuroendocrine malignancies immunoreactive for p16^Ink4A^ showed a decrease in Ki67-positive cells. Quantitative statistical analyses including each histotype and the p16^Ink4A^ status confirmed the independent prognostic role of p16^Ink4A^ expression, being a high-risk indicator in neuroendocrine tumors and a marker of good prognosis in non-neuroendocrine lung malignancies. In this study, we provide circumstantial evidence to suggest that the routinary assessment of p16^Ink4A^ expression using a three-tiered scoring algorithm, even in a small biopsy, may constitute a reliable, reproducible, and cost-effective substrate for a more accurate risk stratification of each individual patient.

## Introduction

Lung cancer represents a heterogeneous group of tumors, embracing distinct entities with different morphologies, molecular features, clinical outcomes, and responses to therapy [1]. The prognosis of these patients is generally poor, with a 5-year overall survival rate of approximately 17% only slightly improved in recent decades [2]. In this era of precision medicine, the search for reliable prognostic markers is of remarkable clinical importance; however, no protein marker has been sufficiently validated for clinical use in lung malignancies, since the results from more than five hundreds indexed studies are inconsistent and/or contradictory [3–5]. To date, tumor histotype and disease stage remain the most important prognostic factors, that drive clinical management and treatment decision [1].

Since p16^Ink4A^ first characterization in the early nineties as a cyclin-dependent kinase inhibitor [6] it has continued to gain widespread importance in a plethora of malignancies, including lung cancer [3, 7–11]. This tumor suppressor protein is encoded by the cyclin-dependent kinase inhibitor 2A (*CDKN2A*, 9p21) and regulates gene expression at different levels by modifying the functional equilibrium of transcription factors, microRNAs, post-transcriptional regulators and ultimately inhibiting transition of the cell cycle from G1 to S phase [12]. To date, the role of *CDKN2A* and its transcript is of burgeoning interest in target therapies. For example, oropharyngeal squamous cell carcinomas overexpressing p16^Ink4A^ respond more favorably to intensity-modulated radiotherapy treatment compared to p16^Ink4A^-negative controls [13]. Furthermore, delivery of the whole p15/p16/p14ARF locus in bacterial artificial chromosomes and induction of p16^Ink4A^ using DNA methyltransferase inhibitors combined with histone deacetylation inhibitors result in the suppression of cell development in human glioma and lung epithelial tumor cells lines, respectively [14, 15]. Overall, ectopic induction of p16^Ink4A^ in cancer cells inhibits cell growth and induces apoptosis and senescence, whereas *CDKN2A* silencing reduces the p53-mediated response to chemotherapeutic agents [16].

The association between p16^Ink4A^ overexpression and patients’ survival in lung cancer has been widely investigated, and there are several lines of evidence to suggest that p16^Ink4A^ could play a part in lung cancer tumorigenesis [3, 17–19]. However, only a handful of previously published studies showed results potentially translatable into clinical practice, highlighting the complexity and the ambiguity underpinning the role of p16^Ink4A^ and p16^Ink4A^-related pathways in lung tumors. At present, the diagnostic, prognostic and predictive values of this essential cell-cycle regulator remain controversial in the lung [20–23]. This could be related to the small sample size of previously published studies focusing only on single major histotypes of lung cancer, often individually, and the lack of correlation with clinical and molecular data. Furthermore, the lack of standardization and reproducibility of previously proposed p16^Ink4A^ scoring systems in lung malignancies, often restricted to a dichotomous record of positive *versus* negative cases might have limited the significance of the previously reported results [17].

In this scenario, the purpose of our study was to investigate the clinical implication of specific patterns of p16^Ink4A^ expression in a large series of lung malignancies using a three-tiered scoring algorithm. Given the histologic and molecular intra- and inter-tumor heterogeneity of lung tumors, and the biologic diversity of each histologic entity, we first sought to define p16^Ink4A^-expression heterogeneity, and subsequently to characterize the prognostic significance of p16^Ink4A^ testing in lung cancers using a semi-quantitative score. Moreover, as a hypothesis-generating aim, we explored the correlation between p16^Ink4A^ status and Ki67 as well as the most common molecular aberrations in well-known targetable cancer genes, such as epidermal growth factor receptor (*EGFR*) and anaplastic lymphoma kinase (*ALK*).

## Materials and Methods

### Patients and tissue specimens

The study was conducted in accordance to local ethical guidelines. After obtaining institutional review board (IRB) approval (San Paolo IRB #10664/13; Fondazione IRCCS Ca’ Granda IRB #179/13), the routinely prepared formalin-fixed paraffin-embedded (FFPE) tissue blocks and the archived slides of consecutive primary lung tumors were retrieved from the archives of the Department of Pathology, A.O. San Paolo Hospital, Milan, Italy and integrated with selected cases retrieved from the archives of the Department of Pathology, Fondazione IRCCS Ca’ Granda—Ospedale Maggiore Policlinico, including rarer histotypes. All patients have provided signed consent forms. Taken together, 502 primary lung tumors, either surgical specimens (n = 383) or core biopsies (n = 119), diagnosed between 2000 and 2015, were included. The study group was composed of 277 adenocarcinomas (ADC), 84 squamous cell carcinomas (SCC), 22 large cell carcinomas (LCC), 47 typical carcinoids (TC), 12 atypical carcinoids (AC), 28 large cell neuroendocrine carcinomas (LCNEC), and 32 small cell carcinomas (SCLC). All cases were centrally reviewed by four pathologists (NF, ADG, EGR, and SF) that performed *ex novo* the histologic classification and pathologic staging, according to the latest editions of the WHO classification of tumors of the lung and of the AJCC staging system, respectively [1, 24]. Specifically, the ADC group included glandular or solid tumors immunoreactive for thyroid transcription factor (TTF1) and/or napsin A; SCCs encompassed keratinizing and non-keratinizing p40-positive, TTF1-negative malignancies; undifferentiated non-small cell malignancies with “null” phenotype (*i*.*e*. lacking TTF1, napsin A, p40, synaptophysin, chromogranin, and CD56 expression) were recorded as LCC. Furthermore, the diagnostic criteria employed for subtyping tumors with neuroendocrine morphology included the number of mitoses per 2 mm^2^, the presence of necrosis, and Ki67 proliferation index [1]. The medical records were subsequently reviewed to obtain patients’ data, including age at diagnosis, gender, smoking history, and survival data. Follow-up data were available for 444 patients until 2015 with a mean follow-up time of 31 months (range 1–144). Clinicopathologic features of the cases included in the study are summarized in Table 1 and detailed in S1 Table. Among 502 cases included in the study, 476 primary lung tumors (either surgical resections or core biopsies) were amenable for multiple sampling and therefore used to construct 15 FFPE tissue microarrays (TMA); the remaining cases were studied on conventional full-face sections. All samples were anonymized prior to processing.

**Table 1:** Clinicopathologic features of the primary lung tumors included in this study.

	ADC n = 277	SCC n = 84	LCC n = 22	TC n = 47	AC n = 12	LCNEC n = 28	SCLC n = 32	Total n = 502

**Male**	166	68	19	10	7	21	19	310
**Female**	111	16	3	37	5	7	13	192
**Smoker**	191/249	77	20	0/2	-	7/7	6/8	301
**Not smoker**	58/249	7	2	2/2	-	0/7	2/8	71
**Grade 1**	21	1	-	47	-	-	-	69
**Grade 2**	144	43	-	-	12	-	-	199
**Grade 3**	112	40	-	-	-	-	-	152
**Grade 4**	-	-	22	-	-	28	32	82
**pT1**	124	25	4	-	-	4/8	3/3	160
**pT2**	117	46	13	-	-	4/8	0/3	180
**pT3**	29	12	4	-	-	0/8	0/3	45
**pT4**	7	1	1	-	-	0/8	0/3	9
**pN-**	182	55	9	44	10	11/21	4/9	315
**pN+**	95	29	13	3	2	10/21	5/9	157

ADC: adenocarcinoma; SCC: squamous cell carcinoma; LCC: large cell carcinoma; TC typical carcinoid; AC: atypical carcinoid; LCNEC: large cell neuroendocrine carcinoma; SCLC: small cell carcinoma.

### Tissue microarrays construction

Using a semi-automatic arrayer (Alphelys Minicore2, Plaisir, France), 1-mm and 3-mm cores were randomly generated based on the amount of tumor tissue available as previously described [25]. Each TMA block contained up to 180 tumor cores with a total number of 2190 spots of neoplastic tissue. For each case, a mean of 4.7 tumor tissue cores (range 2 to 5 cores), including the tumor invasive front, was sampled and one spot of matched non-neoplastic lung tissue for each case was incorporated in the TMAs when available.

### Immunohistochemical analysis

Consecutive 4-microns-thick sections were cut from each FFPE block and subjected to immunohistochemical staining for p16^Ink4A^ (clone DO-7, ID#790–2912, Ventana Medical Systems, Inc., Tucson, AZ) and Ki67 (clone 30–9, ID#790–4286, Ventana Medical Systems, Inc., Tucson, AZ). All non-small cell lung carcinomas included in the TMAs were also tested for *EGFR* status using mutation-specific antibodies for the exon 19 deletion E746-A750 (clone 6B6, ID#9747, Cell Signaling Technologies, MA, USA) and for the exon 21 mutation L858R (clone 43B2, ID#3197, Cell Signaling Technologies, MA, USA). All antibodies were employed according to manufacturers’ instructions as described [26]. Briefly, the sections were subjected to antigen retrieval with Cell Conditioning Solution 1 (CC1, Ventana Medical Systems, Mountain View, CA) and subsequently incubated overnight at 4°C with the primary antibody, washed in 0.1% Tween20/1 × PBS, and then incubated with HRP-conjugated secondary antibodies. Staining was visualized with peroxidase-sensitive Sigmafast 3,3′-diaminobenzidine tablets (DAB; Sigma-Aldrich, St. Louis, MO); sections were counterstained with Mayer’s hematoxylin solution (Amber Scientific, Midvale, WA) and mounted in DPX (BDH, Poole, England).

Immunostaining for p16^Ink4A^ was evaluated independently by two pathologists (NF and SF), and nuclear as well as cytoplasmic staining was considered a positive reaction, as described by Klaes *et al*. [10]. The pattern of p16^Ink4A^ positivity was therefore scored on a semi-quantitative scale for each single tissue core, as follows: negative (less than 1% of positive neoplastic cells), sporadic (isolated positive cells accounting for less than 5% of all neoplastic cells), focal (small clusters of positive cells accounting for less than 25% of all neoplastic cells) and diffuse (more than 25% of positive neoplastic cells) [10]. This system has shown higher levels of reproducibility and accurateness for p16^Ink4A^ scoring in gynecologic malignancies, compared to the semi-quantitative German score [27]. Ki67 was scored independently by three pathologists (NF, ADG and SF) as the percentage of cells with nuclear staining in at least 1000 neoplastic cells randomly selected over 10 high-power (magnification, 40X) fields [28]. The expression of biomarkers for *EGFR* mutations was evaluated as reported elsewhere [29], following the recommendations of the College of American Pathologists, International Association for the Study of Lung Cancer, and Association for Molecular Pathology [30]. For each immunohistochemical analysis, discordant results were resolved on a multi-headed microscope.

### DNA extraction, PCR amplification, and Sanger sequencing

The presence of somatic alterations in *EGFR* exons 19 and 21 was validated by Sanger sequencing analysis in all cases displaying equivocal immunohistochemical stains, as recommended [29, 30]. For this analysis, genomic DNA of each tumor and matched normal tissue was collected from the corresponding FFPE blocks and extracted using the DNeasy^®^ Blood & Tissue Kit (Qiagen, Valencia, CA), according to the manufacturer’s instructions. Primers set to amplify all coding regions of exon 19 (chromosomal position: chr.7: 55,174,722–55,174,820) and exon 21 (chromosomal position: chr.7: 55,191,719–55,191,874) of the *EGFR* gene were employed as described [31]. PCR amplification of 60 ng of genomic DNA was performed using the Ampli*Taq* 360 master mix (Life Technologies, Grand Island, NY). PCR fragments were purified and sequenced on an ABI3730 capillary sequencer as described [32]. Sequences of the forward and reverse strands were analyzed using SeqScape (version 2.5, Applied Biosystems, Carlsbad, CA).

### Fluorescence *in situ* hybridization (FISH)

The presence of the most common inversion event occurring on the short arm of chromosome 2, resulting in the fusion of *ALK* with the echinoderm microtubule-associated protein-like 4 (*EML4*) gene loci, was investigated in all non-small cell lung carcinomas included in the study by means of fluorescence *in situ* hybridization (FISH) [1, 30]. This analysis was performed with a two-color probe mix consisting of BACs for 5′ *ALK* and 3′ *EML4* using validated protocols established at Istituto Nazionale Tumori, Naples, Italy [33]. At least 60 non-overlapping, interphase nuclei of morphologically unequivocal neoplastic cells were analyzed.

### Statistical analysis

Differences in p16^Ink4A^ expression across tumor types were investigated using the Χ^2^ test (MedCalc Software, Acacialaan, Ostend, Belgium). In order to assess the correlation between histotype and p16^Ink4A^ overexpression, a Cox regression analysis was performed assessing first the p16^Ink4A^ status of each case in a dual fashion (negative/positive). A second proportional-hazards regression analysis taking into account the various spectra of p16^Ink4A^ expression was subsequently performed. For the purpose of this work, carcinoids, either typical or atypical, LCNEC, and SCLC were identified as neuroendocrine tumors, whereas ADC, SCC, and LCC were defined non-neuroendocrine carcinomas; survival analysis was performed separately for these two broad groups. Correlation of the protein’s patterns of expression to patients’ overall and disease-free survival was assessed using the Cox proportional-hazards regression model; survival curves were built according to the Kaplan-Meier method (MedCalc software). Patients who died from causes other than lung cancer were excluded for survival analysis. Quantitative analyses were performed using multiple the Cox proportional hazards regression in order to assess the independence of p16^Ink4A^ as a prognostic factor [34]. Probability values (*p*) less than 0.05 were considered statistically significant. Furthermore, to assess the correlation between proliferative indices and p16^Ink4A^ overexpression, a differential Ki67 index (ΔKi67) between p16^Ink4A^-negative and p16^Ink4A^-positive cases was calculated for each histologic subtype, using a combinatorial analysis scheme [35]. This absolute value allows the detection of the variation in the average proliferation indices of each histotype according to p16^Ink4A^ status.

## Results

**Fig 1:**
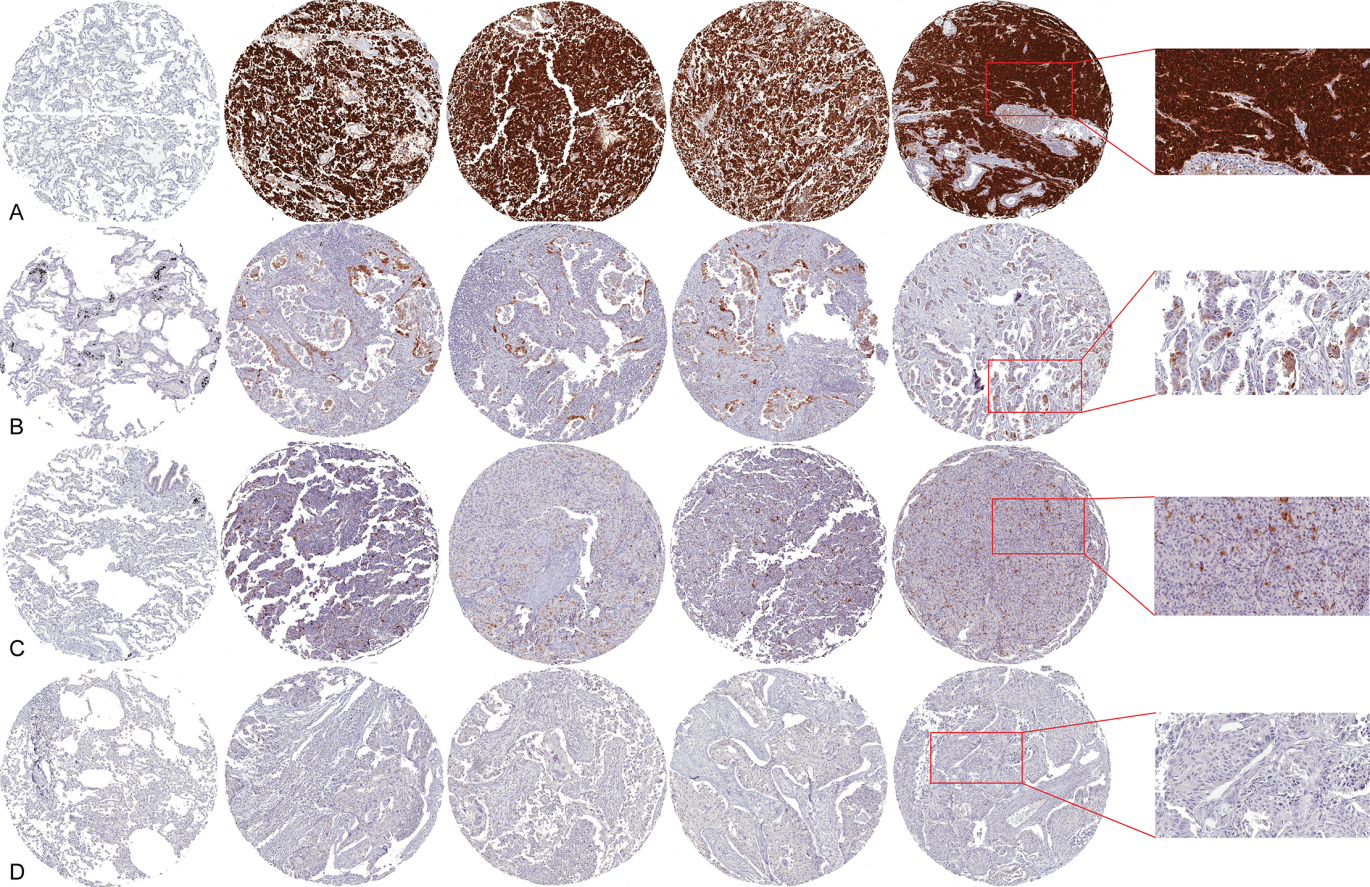
Representative micrographs of p16^Ink4A^ expression patterns in primary lung tumors. For each case, the first core on the left side represents the matched non-neoplastic lung tissue, whereas the following four cores depict different topographic areas of the tumor, including tumor invasive front (original magnification, 5x). The histologic detail of the immunohistochemical analysis for each case can be appreciated in the insets (original magnification, 20x). A. Small cell carcinoma displaying diffuse p16^Ink4A^ immunostain; B. Moderately differentiated (G2) adenocarcinoma showing focal p16^Ink4A^ expression; C. Typical carcinoid with sporadic p16^Ink4A^ expression pattern; D. Poorly differentiated (G3) squamous cell carcinoma negative for p16^Ink4A^.

Taken together, the tumors overexpressing p16^Ink4A^ were 200 (39.8%), including 49 (9.8%) cases with only sporadic immunoreactive cells, 63 (12.5%) showing a focal staining pattern, and 88 (17.5%) that were diffusely positive (Fig 1, Table 2). Matched non-neoplastic lung tissue was universally negative and served as internal negative control.

**Table 2:** Immunohistochemical p16^Ink4A^ status of the primary lung tumors included in this study.

		p16^Ink4A^ overexpression		
	Negative (%)	Sporadic (%)	Focal (%)	Diffuse (%)
**ADC**	166 (59.9)	31 (11.2)	47 (17.0)	33 (11.9)
**SCC**	66 (78.6)	3 (3.6)	6 (7.1)	9 (10.7)
**LCC**	19 (86.4)	-	1 (4.6)	2 (10.0)
**TC**	40 (85.1)	7 (14.9)	-	-
**AC**	7 (58.3)	3 (25.0)	2 (16.7)	-
**LCNEC**	4 (14.3)	3 (10.7)	7 (25.0)	14 (50.0)
**SCLC**	-	2 (6.3)	-	30 (93.7)
	302	49	63	88

ADC: adenocarcinoma; SCC: squamous cell carcinoma; LCC: large cell carcinoma; TC typical carcinoid; AC: atypical carcinoid; LCNEC: large cell neuroendocrine carcinoma; SCLC: small cell carcinoma.

### Primary lung tumors exhibit intra-tumor homogeneous p16^Ink4A^ expression

Intra-tumor heterogeneity analysis of p16^Ink4A^ overexpression was performed for the subgroup of neoplasms incorporated in the TMAs that displayed any pattern of positivity (n = 177). Among them, 171 (96.6%) tumors showed immunoreactive neoplastic cells in each tumor core, whereas heterogeneous p16^Ink4A^ expression across tumor cores was restricted to 6 (3.4%) cases, namely 5 ADCs and 1 SCC, as detailed in Table 3. Of note, the p16^Ink4A^ expression pattern was homogeneous across all cores of the 66 tumors displaying diffuse positivity, suggesting that the analysis of a small area of the tumor is representative of the p16^Ink4A^ of the entire lesion in the vast majority of lung malignancies.

**Table 3:** Intra-tumor heterogeneity analysis of the cases with p16^Ink4A^ overexpression incorporated in the tissue microarrays.

Number of cases	Tumor cores	Cores with p16^Ink4A^ overexpression (%)
32	5	5 (100)
70	4	4 (100)
63	3	3 (100)
6	2	2 (100)
1	5	4 (80)
2	4	3 (75)
1	3	2 (66)
2	5	3 (60)
177		

### p16^Ink4A^ expression in neuroendocrine lung tumors

**Fig 2:**
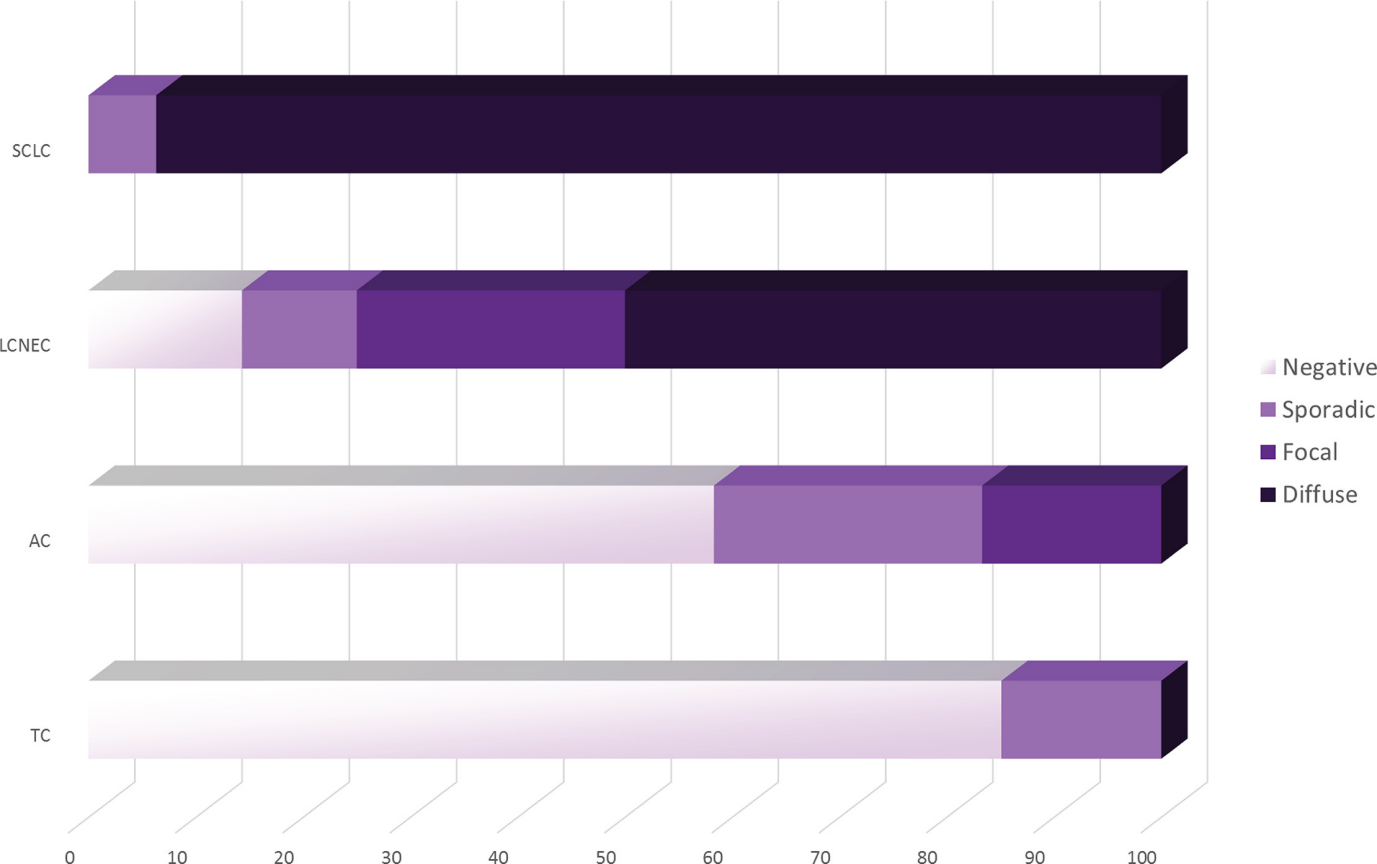
Analysis of p16^Ink4A^ overexpression in neuroendocrine lung tumors reveals an increase in the proportion of positive neoplastic cells within each tumor histotype moving from lower to higher grade malignancies. Each bar represents a histotype, as indicated on the left; the specific patterns of p16^Ink4A^ immunoexpression are color-coded on the basis of the legend on the right. TC: typical carcinoid; AC: atypical carcinoid; LCNEC: large cell neuroendocrine carcinoma; SCLC: small cell carcinoma.

The frequency of neuroendocrine carcinomas expressing p16^Ink4A^ ranged from 14.9% to 100% (Table 2, Fig 2). In particular, only a subset of 7 (14.9%) TCs showed sporadic staining pattern, as exemplified in Fig 1, while 40 (85.1%) tumors were entirely negative. Moreover, 5 (41.7%) AC displayed sporadic or focal p16^Ink4A^ positivity. Taken together, no diffuse expression of the p16^Ink4A^ protein was detected in the carcinoid subgroup, either typical or atypical. Conversely, 14 (50%) LNECs and 30 (93.7%) SCLCs showed a strong and diffuse overexpression of p16^Ink4A^ in all cancer cells (Fig 1), whereas 10 (35.7%) LNECs and 2 (6.3%) SCLCs, displayed sporadic or focal staining patterns. Overall, all SCLCs included in this study displayed immunohistochemical reactivity for p16^Ink4A^, whereas in a subset of LCNECs (n = 4, 14.3%) no expression of p16^Ink4A^ was detected. Therefore, we observed progressive higher p16^Ink4A^-positive tumor cells populations in higher-grade neuroendocrine malignancies (*p* <0.05, Anova) (Fig 2).

**Fig 3:**
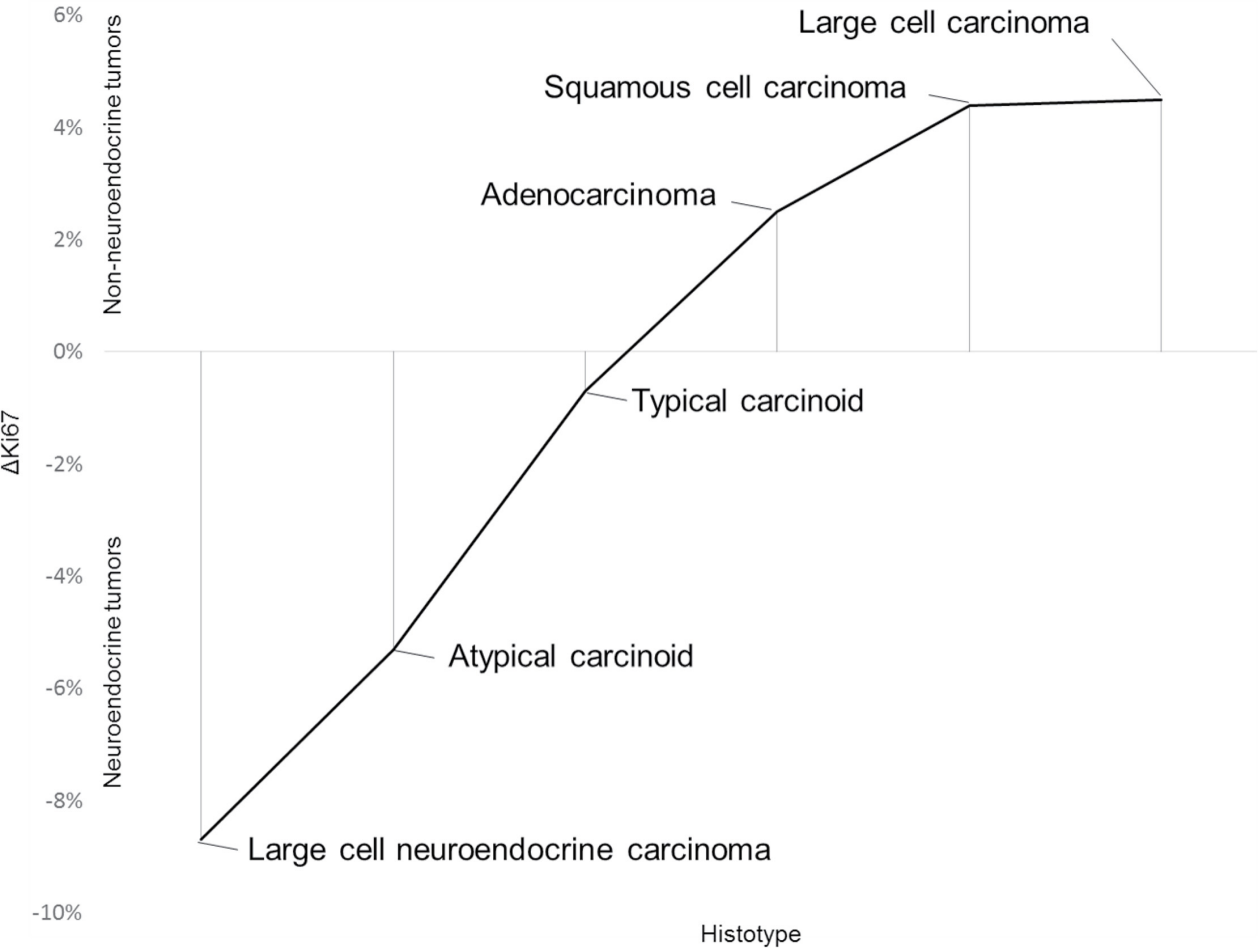
Representation of the differential Ki67 values between p16^Ink4A^-negative and p16^Ink4A^-positive lung tumors. The highest differences in Ki67 indices according to p16^Ink4A^ expression can be observed in the poorly differentiated malignancies (*i*.*e*. small cell carcinomas and large cell carcinomas), with opposite fashions between neuroendocrine and non-neuroendocrine tumors.

In agreement with the results reported above, the correlation between p16^Ink4A^ status and Ki67 index revealed a significantly higher tumor proliferation index in those cases expressing p16^Ink4A^ in at least a subset of neoplastic cells, with a progressive lower ΔKi67 values for TCs, ACs, and LNECs, respectively (Fig 3). A similar association was found for the two SCLCs with sporadic p16^Ink4A^ staining pattern compared to the SCLCs with strong and diffuse positivity (92.5% *versus* 94.4%). No significant correlation was identified between p16^Ink4A^ status and other well-known clinicopathologic parameters such as age, smoke status, stage, and lymph node status (*p* >0.05).

**Fig 4:**
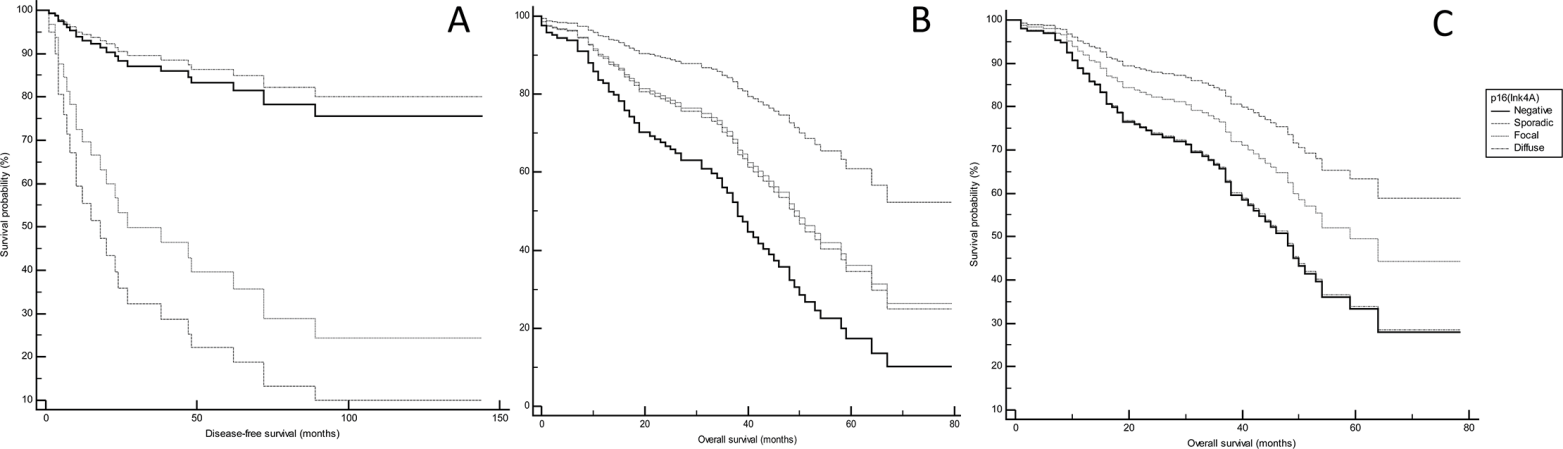
Survival analysis according to p16^Ink4A^ immunohistochemical expression in lung neoplasms. Kaplan-Meier plots reveal that p16^Ink4A^ overexpression is significantly associated with shorter disease-free periods in neuroendocrine tumors (A) and with longer survival times in non-neuroendocrine tumors (B). In particular, even a focal positivity can adversely affect the prognosis of neuroendocrine tumors (A); on the other hand, taking into account tumor stage as a covariate, sporadic and focal positivity cluster together into an intermediate-risk category in non-neuroendocrine malignancies (B), whereas in adenocarcinomas the only focal and diffuse staining patterns are significantly associated with better outcome (C). The survival curves are outlined on the basis of the specific patterns of p16^Ink4A^ immunoexpression, as represented on the right.

p16^Ink4A^ overexpression was significantly associated with a shorter survival time in lung neuroendocrine tumors (HR = 0.08, 95% CI, 0.03 to 0.2, *p* <0.0001) (Fig 4A). Taking into account the various patterns of p16^Ink4A^ expression, a shorter time to recurrence (HR = 0.0826, 95% CI, 0.03 to 0.2, *p* <0.0001) and a decreased overall survival time (HR = 2.48, 95% CI, 1.8 to 3.4, *p* <0.0001) were observed in neuroendocrine malignancies overexpressing p16^Ink4A^, irrespective of staining patterns (S1 Fig). These results were expected, since progressive higher p16^Ink4A^ expression was observed in the aggressive subtypes of neuroendocrine malignancies. However, univariate analyses including each histotype and the p16^Ink4A^ status confirmed that p16^Ink4A^ expression is an independent indicator of poor prognosis in neuroendocrine lung tumors (*p* ranging 0.001 to 0.01).

### p16^Ink4A^ expression in non-neuroendocrine lung malignancies

Overall, the non-neuroendocrine lung carcinomas with p16^Ink4A^ immunoreactivity ranged from 13.6% to 40.1% (Table 2). Among them, 166 (59.9%) ADCs were p16^Ink4A^-negative and 31 (11.2%) ADCs expressed p16^Ink4A^ in less than 5% of the neoplastic cells, whereas 47 (17.0%) tumors showed focal immunoreactivity, as exemplified in Fig 1; for this group, the diffuse pattern of p16^Ink4A^ staining was observed in 33 (11.9%) cases. Nine (10.7%) SCCs displayed sporadic or focal p16^Ink4A^ expression and the same number (10.7%) were diffusely immunoreactive, whereas most of the SCCs analyzed (78.6%) were completely negative (Fig 1). Among LCCs, 1 (4.6%) case displayed a focal staining pattern, 2 (10.0%) cases were diffusely immunoreactive, whereas 19 (86.4%) cases did not express p16^Ink4A^. In contrast to neuroendocrine neoplasms, a significant decrease in the overall p16^Ink4A^ expression was observed for the non-neuroendocrine carcinomas, moving from well differentiated to poorly and undifferentiated entities (p <0.05, Anova) (S2 Fig).

In addition, decreased Ki67 indices were observed in p16^Ink4A^-positive non-neuroendocrine carcinomas, as confirmed by the presence of inverted ΔKi67 values compared to neuroendocrine tumors (Fig 3). Correlation of p16^Ink4A^ status with other clinicopathologic parameters such as age, smoke status, stage, and lymph node status failed to reveal any statistical significance.

Interestingly, in patients with non-neuroendocrine lung malignancies, diffuse or focal p16^Ink4A^-expression was a favorable prognostic factor (HR = 0.08, 95% CI, 0.03 to 0.2, *p* = 0.0123 by Cox proportional-hazards regression analysis). This association was also evident taking into account the tumor pathologic stage as a covariate in the Cox proportional-hazards regression analysis (*p* <0.05), where the survival analysis showed a triple-fashion plot (Fig 4B). Specifically, patients with non-neuroendocrine tumors displaying strong and diffuse p16^Ink4A^ overexpression had a considerable better outcome compared to p16^Ink4A^-negative non-neuroendocrine carcinomas, while patients with tumors showing sporadic and focal staining patterns clustered together in an intermediate-risk group. These data were also confirmed separately for either SCCs (HR = 0.079, 95% CI, 0.03 to 0.211, *p* <0.001) or ADCs, showing decreased overall survival times in patients with tumors displaying patterns of p16^Ink4A^ expression ranging from negative to sporadic (*p* = 0.0123) (Fig 4C). At the molecular level, all lung carcinomas showing diffuse overexpression of p16^Ink4A^ and harboring genetic alterations involving *EGFR* and *ALK* had an excellent outcome, as represented in S2 Table.

## Discussion

In this study, we have characterized the specific spectra of p16^Ink4A^ expression in a large series of neuroendocrine and non-neuroendocrine lung tumors using a three-tiered semi-quantitative score. Our findings provide strong circumstantial evidences to suggest that, despite the well-known histologic and molecular intra-tumor heterogeneity of lung malignancies, the expression of p16^Ink4A^ is homogeneous across each individual neoplastic entity, allowing the analysis of a small area of the tumor, such as a core biopsy, as representative of p16^Ink4A^ status of the entire lesion. Moreover, our observations corroborate the notion that the tumor suppressor protein p16^Ink4A^ plays a dichotomous role in the lung, being a high-risk feature in neuroendocrine tumors and a marker of good prognosis in non-neuroendocrine carcinomas. Finally, we have described a subset of lung carcinomas harboring *EGFR* and *ALK* mutations and overexpressing p16^Ink4A^ that shows better clinical outcome compared to p16^Ink4A^-wild-type tumors.

Spatial heterogeneity analysis of p16^Ink4A^ expression in neuroendocrine and non-neuroendocrine tumors, revealed that p16^Ink4A^ immunoreactivity is homogeneous across different areas of lung tumors, with the same recurring pattern of expression. Since its discovery, the subcellular localization of p16^Ink4A^ has been a matter of great controversy [36]. Hence, although it is intuitive that p16^Ink4A^ acts as a cell cycle inhibitor in the nucleus, there are functional evidences to suggest that the cytoplasmic immunohistochemical expression represent the phenotypic evidence of protein inactivation similar to that observed in other tumor suppressor genes [36, 37]. However, the operational implications of the assessment of defined p16^Ink4A^ expression patterns have not been taken into account in previous studies on lung cancer [4, 20, 22]. In particular, we reasoned that a dualistic immunohistochemical scoring system could be not representative of the actual p16^Ink4A^ status of each individual case, nor sufficiently reproducible. Indeed, it is probable that a considerable proportion of tumors (*i*.*e*. p16^Ink4A^-sporadic and p16^Ink4A^-focal lung neoplasms) has been clustered differently in previous works, determining a substantial heterogeneity in the reported results and therefore leading to a nihilistic and skeptical vision of p16^Ink4A^ as a trustable biomarker in the lung. For this reason, we decided to score the p16^Ink4A^ immunohistochemical nuclear and/or cytoplasmic expression using a semi-quantitative scale, as previously reported for gynecologic malignancies [10]. Hence, we observed that 17.5% of the tumors were strongly and diffusely immunoreactive for p16^Ink4A^, whereas a submodal positivity was restricted to 22.3% tumors. It should be noted that p16^Ink4A^ staining pattern does not necessarily mirror the underlying status of *CDKN2A*; hence, negative immunostaining for p16^Ink4A^ can be seen in some tumors that lack any detectable copy number anomaly in chromosome 9, as assessed by comparative genomic hybridization [38]. Our observations have potential clinical implications, as the assessment of a specific pattern of p16^Ink4A^ expression in a small area of the tumor could provide information on the biological aggressiveness of each individual lung neoplasm.

The assessment of p16^Ink4A^ immunoreactivity in the different histotypes of lung tumor using a semi-quantitative scoring system showed a dichotomous scenario involving neuroendocrine and non-neuroendocrine entities. Our data provide further credence to the notion that these two broad and heterogeneous group of tumors are indeed genetically, and clinically, distinct diseases harboring distinct of mechanisms of p16^Ink4A^ alterations (*i*.*e*. methylation, hemi/homozygous deletion, somatic mutations), as previously reported [18, 19, 39]. In particular, the number of cases overexpressing p16^Ink4A^ even in a submodal proportion of tumor cells, progressively increased from lower- to higher-grade neuroendocrine lung tumors, with percentages varying from 14.9% in TC to 100% in SCLC. Furthermore, the specific fraction of cancer cells immunoreactive for p16^Ink4A^ increased among higher grade neuroendocrine tumor. Our findings confirm that p16^Ink4A^/retinoblastoma (RB) pathway is consistently compromised in LCNEC and SCLC, and less frequently compromised in AC [19]. However, in contrast with previous observations, we observed that in a subset of TC, p16^Ink4A^ display an aberrant pattern in a proportion of tumor cells (*i*.*e*. sporadic staining pattern in TCs and sporadic to focal staining pattern in ACs), suggesting that the immunohistochemical assessment of this tumor suppressor protein should be performed and interpreted carefully in the lung, recording every single positive cell. Intriguingly, p16^Ink4A^ seems to play an opposite role in lung non-neuroendocrine carcinomas, with the percentage of cases displaying the entire spectrum of p16^Ink4A^ positivity significantly higher in well/moderately differentiated tumors (41.3%) compared to poorly differentiated and undifferentiated malignancies (26.1%).These results confirm that *CDKN2A* deregulation through distinct mechanisms, including rare point mutations, promotor methylation and frequent homozygous deletions, is a relatively frequent event occurring in non-small cell lung cancer [18]. A mechanicistic explanation of the clear-cut dichotomous role of p16^Ink4A^ that we observed in lung neoplasms could be provided by recent molecular studies [40]. In particular, our data corroborate previous molecular and phylogenetic findings that could not identify a clonal relationship between neuroendocrine and non-neuroendocrine lung tumors [39, 41]. Hence, the presence of completely different repertoires of somatic genetic alterations between neuroendocrine and non-neuroendocrine tumors of the lung suggests that these two group of neoplasms are, actually, distinct diseases [42, 43]. Furthermore, specific genetic and epigenetic mechanisms are involved in *CDKN2A* instability, according to malignancies originating from distinct anatomical sites [15]. Previous studies demonstrated that promoter silencing of p16^Ink4A^ through methylation could lead to loss of control of the restriction point in the G1 phase of the cell cycle and favor cellular transformation in the lung [19, 40]. Moreover, it has been speculated that the p16^Ink4A^ alteration in lung cancer is a rather early event that may accelerate tumor aggressiveness through inactivation of both the RB and p53 pathways and may link to a poor disease outcome [16, 18, 19, 44]. To this end, large multicentric prospective and functional studies focusing on *CDKN2A* alterations in lung malignancies, including a representative number of rare entities, are warranted. Our observations are consistent with the notion that, while in neuroendocrine tumors p16^Ink4A^ status mirrors the biologic features of higher-grade neoplasms, the expression of this protein in lung non-neuroendocrine malignancies is related to earlier step of tumorigenesis. Based on our results, the assessment of p16^Ink4A^ status, even in a biopsy sample, could improve the characterization of lung tumors.

The correlation analysis between p16^Ink4A^ expression and the proliferative index confirmed the dualistic direction of p16^Ink4A^ immunoreactivity in lung neoplasms. In fact, each neuroendocrine histotype displaying p16^Ink4A^ overexpression had higher tumor proliferative indices, while p16^Ink4A^-positive non-neuroendocrine malignancies showed lower Ki67 rates. These data suggest that the routinary assessment of both p16^Ink4A^ and Ki67 could provide significant information on the specific behavior of each individual histologic subtype of lung malignancies, allowing a fruitful patient’s management.

The contrasting direction of p16^Ink4A^ effect in lung tumors is particularly noticeable in its prognostic value. Despite the great heterogeneity in the previously reported results on the prognostic significance of p16^Ink4A^ in lung malignancies, our analyses demonstrate that p16^Ink4A^ status, if assessed using an accurate semi-quantitative system at the immunohistochemical level, is an independent prognostic factor in lung tumors, being related to poorer prognosis in neuroendocrine entities, and to a more favorable outcome in non-neuroendocrine malignancies. As a matter of circumstance, tumors showing sporadic and focal staining patterns clustered together in an intermediate-risk group in both families of lung tumors. This observation is not trivial, given the evidence that the presence in a small biopsy of a submodal neoplastic population harboring p16^Ink4A^ immunohistochemical overexpression could indicate a worse outcome.

To our knowledge, this is the first study aiming to explore the correlation between p16^Ink4A^ protein expression and mutations in potentially targetable genes. In the small set of cases harboring molecular aberrations in *EGFR* and *ALK* included in this study, the null or heterogeneous p16^Ink4A^ immunohistochemical expression conferred a poor prognosis, suggesting that the assessment of p16^Ink4A^ protein status might help to tailor the prognostic evaluation of *EGFR* or *ALK* aberrant lung cancers.

This study has several limitations. First, given the rarity of ACs, LNECs, and LCCs, we could analyze only a relatively small number of cases. It should be noted, however, that to the best of our knowledge this study represents the first comprehensive analysis of lung neoplasms that provides preliminary data on the prognostic significance of defined patterns of p16^Ink4A^ expression in ACs, LNECs, and LCCs. Second, additional markers mirroring the *CDKN2A* molecular pathways, including cyclin D1 and RB proteins, were not investigated due to the study design focused on the exploration of p16^Ink4A^ routinary testing using a specific scoring system. Our work, however, should be considered as hypothesis generating and validations in wider independent cohorts, with additional and comprehensive molecular data, are warranted.

Despite these limitations, our results suggest that each individual neoplasm of the lung exhibit intra-tumor homogeneous specific patterns of p16^Ink4A^ expression. This tumor suppressor protein plays a contrasting role, being an independent poor prognostic factor in neuroendocrine lung tumors and an independent good prognostic factor in non-neuroendocrine malignancies. In conclusion, p16^Ink4A^ semi-quantitative immunohistochemical analysis is a reliable, reproducible, and cost-effective testing that can potentially be employed to improve the characterization of lung neoplasms, allowing a more accurate patient’s management. Large clinical prospective studies coupled with immunohistochemical and molecular analyses are warranted to delineate the ramifications of our findings for both pathologist and oncologists.

## Supporting Information

S1 Fig Kaplan-Meier curves of lung neuroendocrine tumors for overall survival according to the different patterns of p16^Ink4A^ expression. (PPTX)

S2 Fig p16^Ink4A^ patterns of expression in non-neuroendocrine lung tumors according to the percentage of positive cases, clustered in higher- and lower-grade diseases. (DOCX)

S1 Table Clinicopathologic features of the cases included in this study. (XLSX)

S2 Table Clinicopathologic features, molecular characteristics and clinical outcome of patients with somatic genetic aberrations included in this study. Highlighted in bold the cases displaying diffuse p16^Ink4A^ staining patterns.
(XLSX)
