# Supplementary material for: The Contrasting Role of p16Ink4A Patterns of Expression in Neuroendocrine and Non-Neuroendocrine Lung Tumors: A Comprehensive Analysis with Clinicopathologic and Molecular Correlations
Source: PLoS One. 2015 Dec 16;10(12):e0144923. doi: 10.1371/journal.pone.0144923 (PMC4684221; doi:10.1371/journal.pone.0144923)
Supplement: S1 Fig — (PPTX) [file pone.0144923.s001.pptx]

## Slide 1
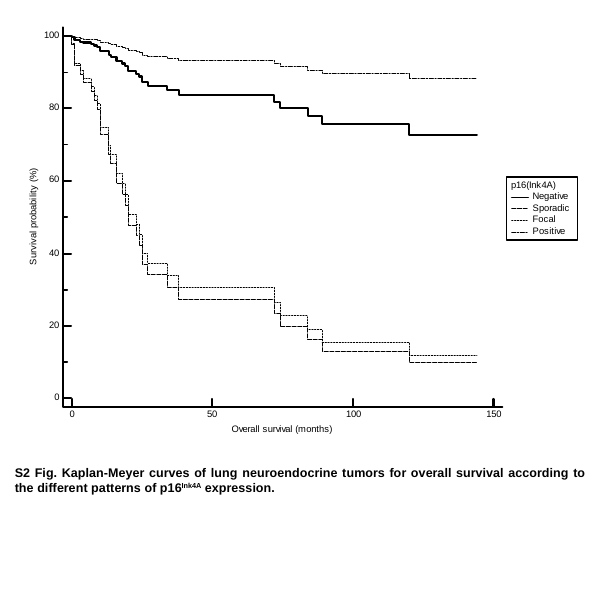

S2 Fig. Kaplan-Meyer curves of lung neuroendocrine tumors for overall survival according to the different patterns of p16Ink4A expression.
