# Supplementary material for: The Contrasting Role of p16Ink4A Patterns of Expression in Neuroendocrine and Non-Neuroendocrine Lung Tumors: A Comprehensive Analysis with Clinicopathologic and Molecular Correlations
Source: PLoS One. 2015 Dec 16;10(12):e0144923. doi: 10.1371/journal.pone.0144923 (PMC4684221; doi:10.1371/journal.pone.0144923)
Supplement: S2 Fig — (DOCX) [file pone.0144923.s002.docx]

**S2 Fig. p16^Ink4A^ patterns of expression in non-neuroendocrine lung tumors according to the percentage of positive cases, clustered in higher- and lower-grade diseases.**


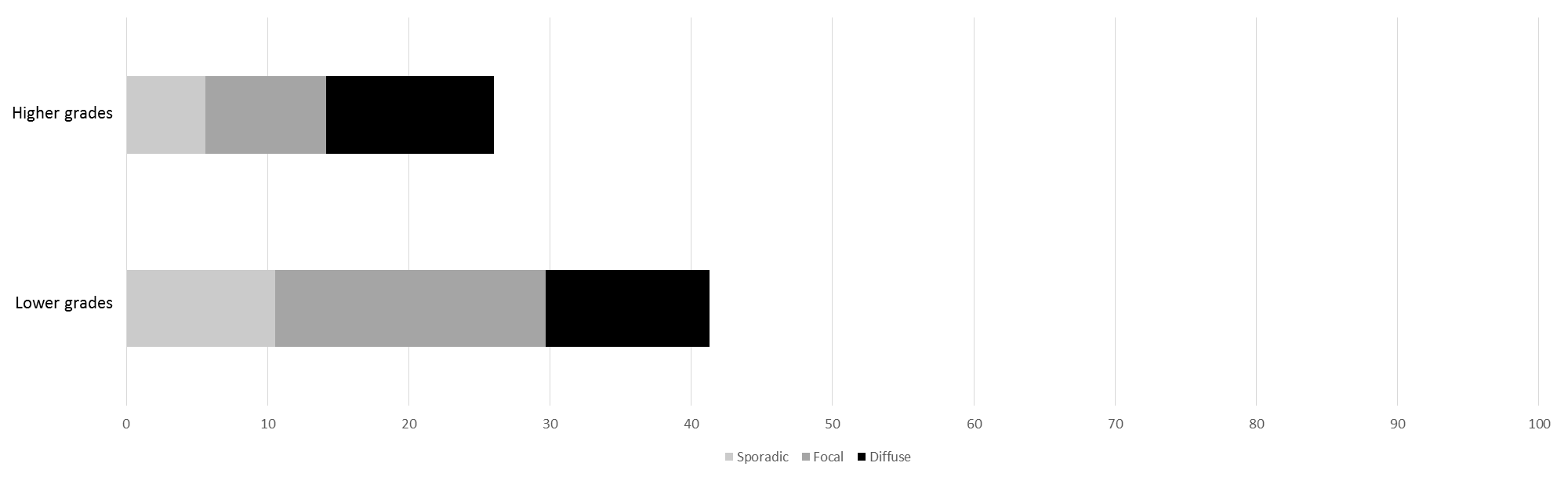


Lower grades, well (G1) and moderately (G2) differentiated neoplasms; Higher grades, poorly differentiated (G3) and undifferentiated (G4) neoplasms.
